# Effect of immediate photobiomodulation on pain and inflammation after oral biopsy: A randomized clinical study

**DOI:** 10.1007/s00784-026-06791-1

**Published:** 2026-02-24

**Authors:** Yolanda Collado-Murcia, Francisco Parra-Perez, Pia López-Jornet

**Affiliations:** 1 Department of Dermatology, Stomatology, Radiology and Physical Medicine, Faculty of Medicine, Regional Campus of International Excellence Campus Mare Nostrum, University of Murcia and Biomedical Research Institute of Murcia (IMIB), Ctra. Madrid-Cartagena, s/n, Murcia, El Palmar, 30120 Spain; 2 Regional Campus of International Excellence ’Campus Mare Nostrum, Murcia, Spain; 3 Hospital Morales Meseguer Clínica Odontológica, Medicina oral Marques Velez S/N, Murcia, 3008 Spain

**Keywords:** Oral biopsy, Pain, Photobiomodulation, Swelling

## Abstract

**Objective:**

To evaluate whether photobiomodulation (PBM) reduces postoperative pain and inflammation in patients undergoing a soft tissue oral biopsy, compared with a sham treatment.

**Materials and methods:**

A prospective, randomized controlled clinical study was conducted. Oral mucosal biopsies were performed using a standardized protocol. Group allocation was carried out using simple randomization. Participants were assigned to two groups: active PBM (*n* = 31) and simulated (sham) PBM (*n* = 31). A single intraoral PBM session (wavelength 940 nm, output power 0.5 W) was applied immediately after the biopsy procedure.

**Results:**

No statistically significant differences were observed between the groups with respect to pain or inflammation reduction during the 7-day follow-up period. Both variables decreased over time in both groups, with no significant group effect or group–time interaction effect (*p* = 0.279 and *p* = 0.220, respectively).

**Conclusions:**

A single session of PBM applied immediately after a soft tissue oral biopsy did not demonstrate additional benefits compared with sham treatment in reducing postoperative pain or inflammation over a 7-day period. Further studies using optimized PBM protocols are needed to better define its potential role in this clinical setting.

## Introduction

 Biopsies are the gold standard procedures for the diagnosis of many oral lesions. These are minor surgical interventions in which a tissue fragment is removed to examine it histologically in order to obtain a complete diagnosis along with a clinical diagnosis [1]. For this, different instruments must be used, and there are different classifications according to the technique, extent, and amount of tissue removed. A biopsy in the oral cavity can provoke undesirable secondary effects such as pain and inflammation [2], aside from producing different levels of stress and uncomfortableness in the patient [3, 4]. This anxiety towards oral and dental procedures can delay or hinder diagnosis [5, 6]. In addition, high levels of anxiety towards dental treatments have been related to increases in the feelings of pain of the patient and delays in recovery [7]. To reduce these levels of stress, diverse techniques are being used, with non-pharmacological ones increasing in popularity [8]. The use of laser-based systems are being implemented after oral interventions, as photobiomodulation (PBM) produces beneficial effects in the management of inflammation and pain, and therefore, the comfort of patients and their level of anxiety [9]. PBM is associated with an increase in vascular microirrigation [10] with an increase in vascularization observed in the surgical area along with the presence of newly-formed blood vessels and a high proliferation of cells [11, 12]. This therapy is being used as a coadjuvant after extractions of third molars [13, 14], for the prevention of oral mucositis in cancer patients along with other treatments for the management of pain [15, 16], as well as patients with burning mouth syndrome [17], or to improve trismus in patients with temporomandibular joint disorders [18]. However, no studies have applied PBM after oral biopsies in soft tissue. The aim of the present study was to assess if photobiomodulation reduces postoperative pain and inflammation of patients subjected to an oral biopsy in soft tissue, as compared with a simulated treatment.

## Materials and methods

A prospective, randomized, exploratory clinical study was conducted at the University Dental Clinic of the Department of Oral Medicine, Morales Meseguer Hospital (Murcia, Spain), between January 2023, and June, 2025. All eligible consecutive patients were invited to participate.

The study protocol adhered to the ethical principles of the Declaration of Helsinki. The project was approved by the Bioethics Committee of the University of Murcia (ID: 2934/2020). All the participants signed an informed consent after their inclusion.

### Participants

#### Inclusion criteria

Patients older than 18 years of age, who required a biopsy in soft tissue in the oral cavity according to a diagnostic or therapeutic indication, who provided their written informed consent, after receiving all the information about the procedure.

#### Exclusion criteria

Patients not apt for minor surgery according to the ASA classification; presence of decompensated systemic diseases; **i**mmunosuppression, radiotherapy, **p**regnancy; or severe mental disorders that would prevent cooperation or follow-up.

**Fig. 1:**
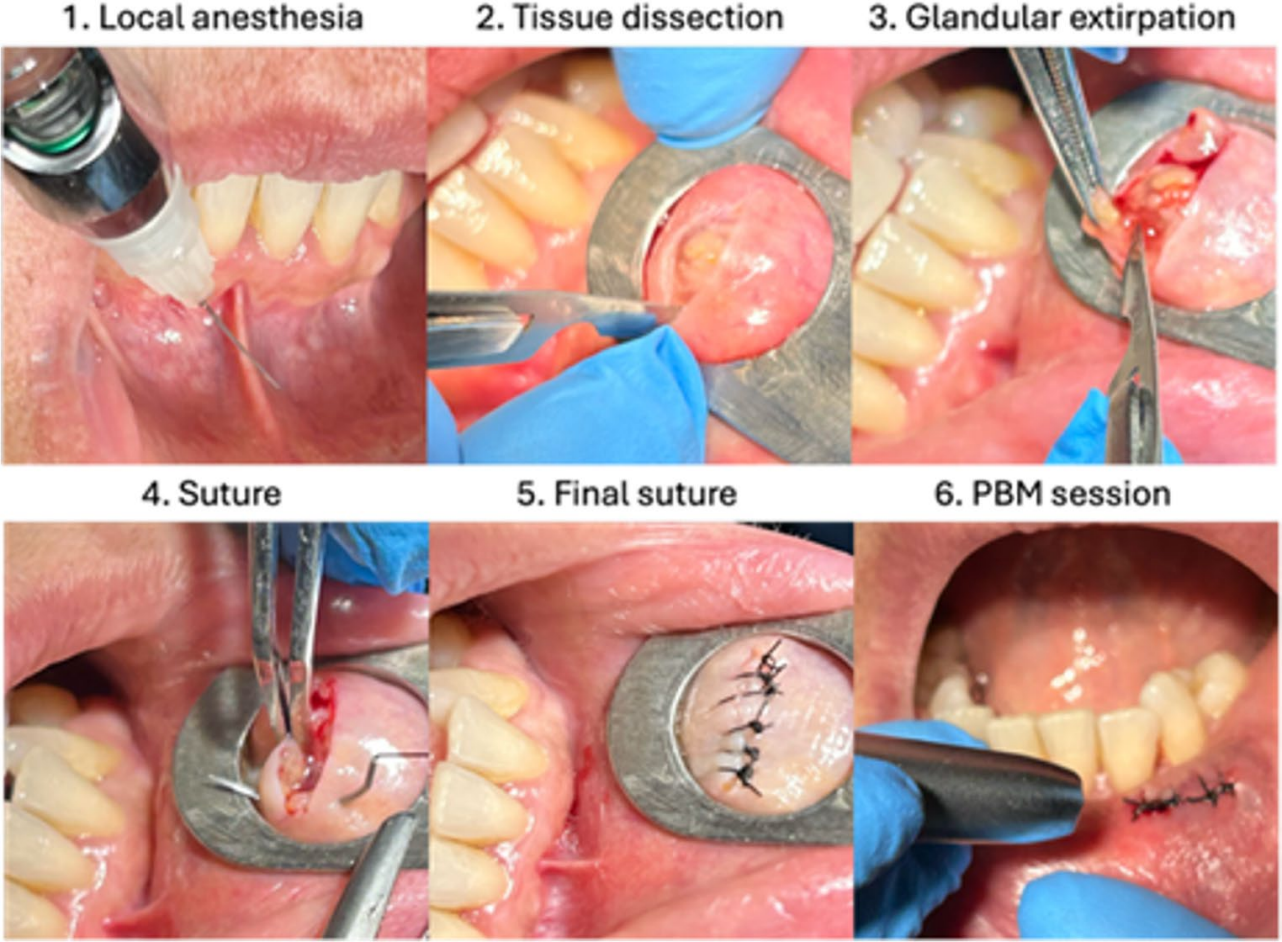
Minor salivary gland biopsy in the lip for diagnosis of Sjögren’s Syndrome with PBM session (test group)

**Fig. 2:**
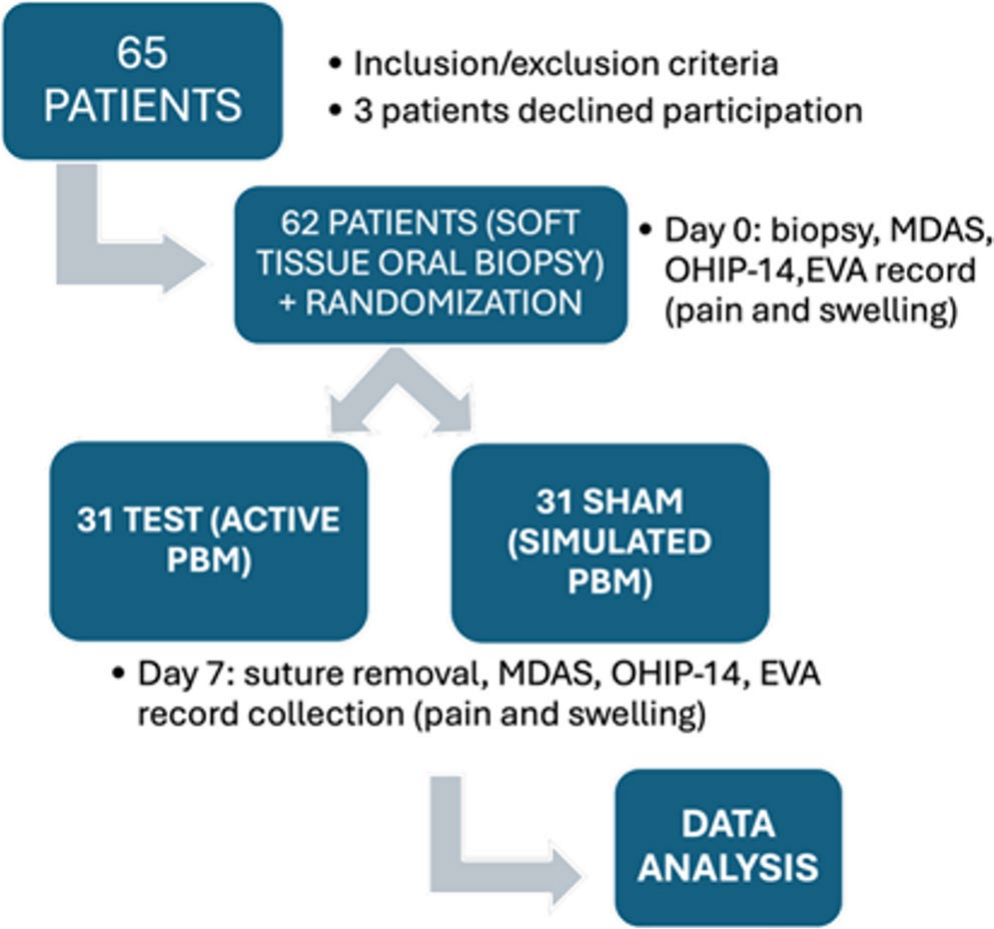
Flow diagram of the study

A prospective, randomized, exploratory and partially double-blinded clinical study was performed. Both the patient and evaluator were unaware of the type of treatment assigned (active or simulated PBM). The operator, due to technical reasons, knew about the assignment, but did not participate neither in the collection or analysis of the data. Group allocation was performed using the OxMaR program (Oxford Minimization and Randomization) [19]. The sample size was determined according to the availability of the cases during the study period and was considered adequate for an exploratory analysis. The following data were recorded: age, sex, habits, location of the biopsy, type of scalpel, oral hygiene, duration of the surgery (min), and initial The Modified Dental Anxiety Scale (MDAS) was used to assess dental anxiety and the Oral Health Impact Profile (OHIP-14Sp) to evaluate oral health–related quality of life. All the participants were subjected to a conventional soft-tissue biopsy under local anesthesia(Fig. 1). Immediately after, the laser procedure was applied according to the group assignment (Fig. 2) :

- Test group (active PBM): a single intraoral laser session with an EPIC X Biolase diode laser (BIOLASE, Inc., USA) wavelength 940 nm, 0.5 W, 15 J/cm², for 10–30 s (adapted to the size of the wound) at a distance of approximately 1 mm using a sterile tip. The parameters were selected according to the recommendations described by Cronshaw et al. [20] for biostimulation and management of pain/inflammation. (Table 1)
- Control group (simulated PBM): identical procedure without an effective emission (sham).

**Table 1:** Photobiomodulation (PBM) protocol parameters

Parameter	Active PBM group
Laser device	EPIC X diode laser (BIOLASE, Inc., USA)
Wavelength	940 nm
Emission mode	Continuous wave
Output power	0.5 W
Energy density (fluence)	15 J/cm²
Application time	10–30 s (adapted to wound size)
Distance from tissue	Approximately 1 mm
Application technique	Intraoral, non-contact
Tip	Sterile disposable tip
Number of sessions	1
Timing of application	Immediately after tissue sampling
Treated area	Biopsy wound area

After the intervention, both groups received standard postoperative instructions: no rinsing the mouth or spitting on the same day, avoidance of smoking, maintenance of careful oral hygiene, and a soft food diet. No routine postoperative analgesic prescription was provided. Patients were allowed to take rescue analgesics if required; however, analgesic consumption was not systematically recorded.

### Procedure

Conventional soft-tissue biopsies were performed using a cold scalpel or punch, depending on clinical indication. Suturing was performed when considered necessary according to wound size and clinical judgment; therefore, not all cases required sutures, particularly punch biopsies. Photobiomodulation was applied only after tissue sampling and therefore did not interfere with histopathological diagnosis.

#### Visit 1 (Day 0 – Biopsy)

The biopsy was performed, the baseline data were collected, and the assigned intervention was applied (active or simulated PBM). The patient was given a notebook to record pain and inflammation every day, through a **visual analog scale (VAS)**, during the following 7 days, with uniform instructions.

#### Visit 2 (Day 7)

The sutures were removed, the VAS scales were collected, and the **MDAS** (dental anxiety) and **OHIP-14Sp** (quality of life) questionnaires were repeated. The possible local complications were recorded (dehiscence, hematoma, bleeding, or infection).

## Variables and assessments

### Main variables

- Postoperative pain and inflammation, assessed with a VAS scale (0–10), where 0 indicates absence and 10 maximum intensity. The pain and inflammation were recorded with a self-administered scale (VAS), reducing the risk of bias by the evaluator. **Perceived (subjective) inflammation was defined as patient-reported swelling** and assessed using a Visual Analogue Scale (VAS, 0–10), where 0 represented no swelling and 10 the maximum imaginable swelling. This method has been previously used to evaluate post-surgical oral inflammation [21].

### Complementary evaluations

- **Modified Dental Anxiety Scale (MDAS)**: 5-item instrument, each scored from 1 to 5 (total range 5–25). The MDAS was applied at the start and end of the follow-up. Classification:
  - 5–9: low or normal anxiety.
  - 10–12: moderate anxiety.
  - 13–14: high anxiety.
  - ≥ 15: possible dental phobia.
- **OHIP-14Sp questionnaire (Oral Health Impact Profile**,** abbreviated version)**: instrument validated in Spanish by Montero et al. [22], composed of 14 items distributed in seven dimensions. Each item is scored from 1 (never) to 5 (very often), with range of 14–70 points. The higher scores indicated a greater negative impact on oral health in quality of life. This questionnaire was applied at the start and after seven days.
- Postoperative complications were recorded (dehiscence, hematoma, bleeding, and infection).

### Statistical analysis

The SPSS software (version 29.0) was used. Given the intermediate size of the sample (*n* = 62), a normality study of the variables was necessary (Kolmogorov-Smirnov test), which rejected the hypothesis that the variables are normal, and therefore the objectives were addressed using a non-parametric approach. The aim of the inferential analysis was to determine the existence or not of significant differences between the control and test groups. For pain and inflammation, the Brunner-Langer model was estimated with the intra-subject factor being time (T1 to T7) of measurement, and the inter-subject factor being the patient group, in order to study the effect of these factors on non-normal variables. In the case of a binary outcome, a binary logistic regression with OR (odds ratio) estimations was used. To assess the homogeneity of the groups with respect to the pre-surgery variables, the Chi^2^ and Mann-Whitney tests were used. The level of significance used in the analyses was 5% (α = 0.05).

## Results

### Descriptive analysis

The sample was composed of 62 individuals who were subjected to a soft tissue biopsy in the oral cavity. The mean age of the participants was 55.7 years old, with a standard deviation of 17.4 years and a range from 18 to 84 years old. About a fourth of the participants consumed alcohol and a third smoked (Table 2). In addition, 79% of the sample showed bad mouth hygiene. With respect to anxiety, in the pre-surgery assessment, 86% had mild-none anxiety, 11% moderate, 2% high, and 2% severe. As for quality of life, a mean of 4.3 was observed in the OHIP-14Sp scale, with a standard deviation of 11.7 units. Also, 64.5% of the lesions analyzed were potentially malignant oral lesions, 19.4% of the biopsies performed were done so to complete the Sjögren syndrome or amyloidosis diagnosis, and the remaining 16.1% were catalogued as other tumorous lesions in the oral cavity.

**Table 2:** Description of the most relevant data from the study

	Group		*p*
	Control/SHAM (*n* = 31)	Test (*n* = 31)	
Gender			0.444
Male	12 (38.7%)	16 (51.6%)	
Female	19 (61.3%)	15 (48.4%)	
Age (years)	55.8	55.5	0.961
Duration of surgery (minutes)	10.5	12.6	0.017
Biopsy location			0.330
Gum	17 (54.8%)	12 (38.7%)	
Tongue	4 (12.9%)	5 (16.1%)	
Lower lip	8 (25.8%)	7 (22.6%)	
Palatal	1 (3.2%)	1 (3.2%)	
Buccal mucosa	1 (3.2%)	6 (19.4%)	
OHIP-14Sp TOTAL (Day 0)	4.5	4.1	0.163
MDAS TOTAL (Day 0)	2.4	4.2	0.793
Suture			0 0.444
Yes	16 (51.6%)	12 (38.7%)	
No	15(48.4%)	19 (61.3%)	
Type of scalpel			0.730
Cold scalpel	27 (89.1%)	25 (80.6%)	
Punch	4 (12.9%)	6 (19.4%)	
Hygiene			1
Good hygiene	7 (22.6%)	6 (19.4%)	
Poor hygiene	24 (77.4%)	25 (80.6%)	

### Pain and inflammation analysis

The pain significantly decreased over time (*p* < 0.001) and equally in both groups (*p* = 0.279). There was no significant evidence in that the mean level of pain was different between both groups (*p* = 0.993). In the sham group, significant differences were found between day 3 and day 1, and in the test group between many of the days (Tables 3 and 4).

**Table 3:** Changes in pain in the Sham group

	Day 1	Day 2	Day 3	Day 4	Day 5	Day 6
**Day 2**	**1**					
**Day 3**	0.033*	0.167				
**Day 4**	0.067	0.276	1			
**Day 5**	0.119	0.244	1	1		
**Day 6**	0.208	0.316	1	1	1	
**Day 7**	0.303	0.373	1	1	1	1

*p<0.05; **p<0.01; ***p<0.001

**Table 4:** Changes in pain in the test group

	Day 1	Day 2	Day 3	Day 4	Day 5	Day 6
**Day 2**	0.827					
**Day 3**	0.062	0.077				
**Day 4**	0.021	0.013*	0.516			
**Day 5**	0.033*	0.033*	0.727	1		
**Day 6**	0.022*	0.021*	0.407	1	1	
**Day 7**	0.106	0.006**	0.236	0.931	0.863	1

*p<0.05; **p<0.01; ***p<0.001

Inflammation significantly decreased over time (*p* < 0.001) and similarly in both groups (*p* = 0.220). There was no significance evidence that the mean level of inflammation was different in both groups (*p* = 0.995). At the start, both groups had similar levels of inflammation, but in the test group it decreased slower. In the sham group, no significant differences were observed between the different days. In the test group, significant differences were observed between day 1 and days 4–7 and between day 2 and days 3–7. (Tables 5 and 6).

**Table 5:** Changes in inflammation in the Sham group

	Day 1	Day 2	Day 3	Day 4	Day 5	Day 6
**Day 2**	1					
**Day 3**	0.148	0.089				
**Day 4**	0.068	0.104	1			
**Day 5**	0.149	0.192	1	1		
**Day 6**	0.131	0.143	1	1	1	
**Day 7**	0.187	0.227	1	1	1	1

*p<0.05; **p<0.01; ***p<0.001

**Table 6:** Changes in inflammation in the test group

	Day 1	Day 2	Day 3	Day 4	Day 5	Day 6
**Day 2**	1					
**Day 3**	0.086	0.047*				
**Day 4**	0.012*	0.013*	1			
**Day 5**	0.011*	0.009**	0.294	0.610		
**Day 6**	0.032*	0.038*	0.475	1	1	
**Day 7**	0.009**	0.008**	0.091	0.169	0.307	0.309

^*p<0.05; **p<0.01; ***p<0.001^

- Analysis of pain and inflammation according to hygiene.

Both pain and inflammation significantly decreased over time (*p* = 0.009) (*p* = 0.006) and in a similar manner, independently of hygiene (*p* = 0.287) (*p* = 0.615). There was no significant evidence that the mean level of pain (*p* = 0.926) nor inflammation (0.247) was different according to hygiene.

- Analysis of pain and inflammation according to location and type of scalpel used.

**Fig. 3:**
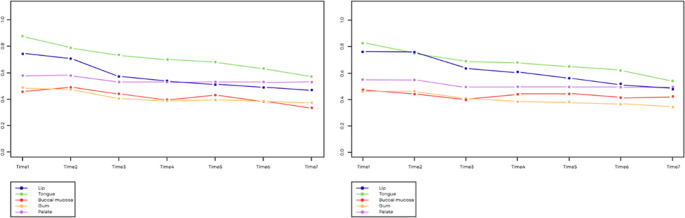
Changes in pain (left) and inflammation (right) according to the location of the lesion

In the sham group, most had lesions in the gums and lips, while in the test group, there was a greater distribution between both locations. Pain significantly decreased over time (*p* < 0.001) and similarly, independently of the location of the lesion (*p* = 0.144). There was no significant evidence that the mean level of pain was different according to location (*p* = 0.389). In addition, inflammation significantly decreased over time (*p* < 0.001) and with a marginal significance, in a different manner according to the location of the lesion (*p* = 0.061). Certain locations, such as the lips and tongue, showed a significant initial inflammation, as observed in Fig. 3. The results suggest a different level of inflammation according to location (*p* = 0.061), although independent from the assessment day.

On the other hand, there was no significant evidence that the mean level of pain was different according to the scalpel used (*p* = 0.793) or the level of inflammation (*p* = 0.787).

- Analysis of pain and inflammation according to anxiety and quality of life.

There was no significant evidence that the mean level of pain was different according to anxiety (*p* = 0.05), nor daily differences in the decrease in pain due to anxiety (*p* = 0.477). As for quality of life, pain significantly decreased over time (*p* = 0.002) and in a different manner according to the OHIP-14sp score (*p* = 0.024). The patients with a better quality of life (score = 0) experienced pain reduction with a stronger slope.

There was no significant evidence that the level of mean inflammation was different according to anxiety (*p* = 0.384). And neither were daily differences in the decrease in inflammation due to anxiety (*p* = 0.711). The patients with an initially better quality of life experienced a marked reduction in the degree of inflammation, just as it was observed with pain (*p* = 0.016).(Tables 7 and 8).

**Table 7:** MDAS scale, day 0 (T1: basal), day 7 (T2: post-treatment) and difference between them and OHIP14-Sp scale, day 0 (T1: basal), day 7 (T2: post-treatment) and differences between them

	GROUP				
		TOTAL	Control/SHAM	Test	*p*-value
MDAS BASAL (T1)	**Total**	62(100%)	31 (100%)	31 (100%)	0.477
	**Mild-none (0–8)**	53(85.6%)	29 (93.5%)	24(77.4%)	
	**Moderate (9–12)**	7 (11.3%)	2(6.5%)	5 (16.1%)	
	**High (13–14)**	1(1.6%)	0 (0.0%)	1 (2.3%)	
	**Severe (> 15)**	1 (1.6%)	0 (0.0%)	1 (3.2%)	
MDAS POST (T2)	**Total**	62 (100%)	31(100%)	31 (100%)	
	**Mild-none (0–8)**	58 (93.5%)	30 (96.8%)	28 (90.3%)	
	**Moderate (9–12)**	4 (6.5%)	1 (3.2%)	3 (9.7%)	
OHIP14-SP (T1)	**N**	62	31	31	0.164
	**Mean**	4.3	4.5	4.1	
	**Standard deviation**	11.7	13.5	9.9	
OHIP14-SP (T2)	**N**	62	31	31	
	**Mean**	6.1	7.4	4.8	
	**Standard deviation**	12.0	13.8	9.8	

### Analysis of the postoperative complications

The same rate of appearance of complications was observed in both groups: 22.6%. Likewise, the duration of the surgery, hygiene, and the type of scalpel used were not associated with the risk of having complications. An association with a marginal significance was found between the variable location and the probability of having some complication; when the location of the biopsy was on the lips, it was multiplied by 4 with respect to the reference (gums). The likelihood of having complications when the location was the gums was 13.8%, while when the location was the lips, it was 40%.

**Table 8:** Most frequent complications by lesion location: results of the simple logistic regression model: odds ratio (OR) and 95% confidence interval

	OR	95% CI	*p*-value
LOCATION			
Gum	1		
Tongue	3.12	0.51–18.36	0.200
Lower lip	4.17	0.97–19.80	0.058
Buccal mucosa	1.04	0.05–8.86	0.973
*p<0.05; **p<0.01; ***p<0.001			

## Discussion

Pain perception is inherently subjective and may vary according to individual thresholds, psychological factors, and previous experiences. The results of the present study indicate that a single session of PBM applied immediately after an oral biopsy did not significantly reduce postoperative pain or inflammation during the 7-day follow-up period. This finding suggests that although PBM has a biostimulant potential, as shown in other contexts (mucositis, wisdom tooth extraction), its isolated and immediate application may be insufficient for inducing a sustained biological effect as most positive outcomes reported in the literature rely on multi-session protocols.

The progressive decrease in pain and inflammation observed in both groups is consistent with the natural healing process, which is usually rapid and uncomplicated. In the study by Emperumal et al. [23], the procedure was reported to be well tolerated and associated with minimal levels of anxiety or fear. In addition, the postoperative pain after an oral biopsy was generally low, with a tendency to disappear after the first week [21].

The findings coincide with the recent literature, which showed that an oral biopsy is a safe and low impact procedure with a fast recovery. Lajolo et al. [24] reported that only 27–30% of patients experienced clinically relevant pain or discomfort (NRS ≥ 4; OHIP-14Sp ≥ 20) six hours after the procedure, with a significant reduction observed after seven days. These authors found a correlation between a worse quality of life and a higher degree of pain perceived, but without an influence from the type of technique or the sample size.

Cohen et al. [25], in a retrospective study with 695 patients, reported an incidence of chronic pain after the biopsy of only 0.57%, with all the cases being mild and self-limiting. Oral biopsies produce a mild tissue trauma, and they are rarely associated with nerve complications. Therefore, the potential impact of immediate PBM is difficult to detect in procedures characterized by minimal pain and rapid recovery.

On the other hand, studies on PBM in more invasive procedures have shown favorable results. In procedures such as third molar extraction, chemotherapy-induced oral mucositis, or periodontal surgery, multi-session PBM protocols using wavelengths between 660 and 980 nm have been associated with significant reductions in pain and edema [26, 27]. These differences could be attributed to the greater surgical trauma of these procedures, together with the repeated use of the laser.

Until today, no consolidated studies on PBM applied to conventional oral biopsies have been found. The only previous study that is partially comparable is the one by Palaia et al. [2], in which the biopsy was performed with a surgical laser, a technique that produces a tissue response that is different due to its photothermal and hemostatic effect. As a result, the direct comparison is limited. Our results, along with those by Emperumal [23] and Lajolo [24], contribute to the definition of the real clinical profile of the conventional biopsy with a cold scalpel: a well-tolerated procedure with minimum inflammation and self-limiting pain. The use of the cold scalpel in our study ensures comprehensive histological margins and a predictable inflammatory response. On the other hand, Lazzarotto et al. [28], in a review of 1089 biopsies, showed that the size of the fragment –rather than the cutting technique- was the main determining factor of a concluding diagnosis. In their series, samples ≥ 10 mm doubled the probability of obtaining a definite diagnosis. This finding reinforces the importance of prioritizing the representativeness of the tissue as opposed to considerations of postoperative comfort. The low baseline levels of pain and inflammation associated with conventional oral biopsy may limit the ability to detect additional clinical benefits.

The assessment through the MDAS and OHIP-14Sp scales did not show significant correlations between pre-surgery anxiety, quality of life, and postoperative symptoms. This finding coincides with that described by Emperumal et al. [23] who found mean anxiety/fear scores of 1/10 a week after the procedure, indicating that the emotional memory of the biopsy is mild and transitory. Although in more extensive surgeries pre-surgery anxiety can amplify the perception of pain, in minor procedures such as an oral biopsy, the psychological impact seems to be marginal. From the clinical perspective, the results support the safety of immediate PBM after an oral biopsy, although without demonstrating significant analgesic or anti-inflammatory benefits. The technique is presented as an innocuous alternative that is potentially useful for improving the subjective perception of comfort, especially in patients who are anxious or with a history of exaggerated pain during dental procedures. However, its systematic application does not seem to be justified based on current evidence.

### Limitations and future perspectives

To date, no clinical studies have examined the immediate application of photobiomodulation after a conventional oral biopsy; for this reason, the results should be interpreted with caution and within the context of an exploratory study. The main limitations include the lack of a priori sample size calculation and the predominantly subjective assessment of pain and inflammation, without the inclusion of objective inflammatory markers. A single PBM session was intentionally applied to mirror routine clinical practice, as oral biopsies are usually performed on a single visit. Postoperative analgesic intake was not systematically recorded and, together with the absence of systematic classification of biopsy type (incisional versus excisional), may in part have influenced the detection of mild clinical effects. Additionally, inclusion of incisional biopsies with remaining lesion may have introduced some heterogeneity in postoperative outcomes; however, evaluation was focused on surgical site healing, postoperative complications, and patient-reported symptoms. Nevertheless, these findings provide relevant preliminary information and support the need for future studies with larger samples, objective outcome measures, and optimized PBM protocols.

## Conclusion

The results of this study confirm that conventional oral biopsy is a safe and well-tolerated procedure, associated with rapid recovery and low levels of postoperative pain and inflammation. The immediate application of a single photobiomodulation session did not demonstrate significant benefits in reducing pain or inflammation compared with sham treatment. Nevertheless, PBM was well tolerated and no adverse effects were observed. Within the context of this exploratory study, routine use of PBM after conventional oral biopsy cannot currently be recommended, and further studies with larger samples and optimized protocols are warranted to better define its clinical role.

## Data Availability

The data presented in this study are available on request from the corresponding author.
